# Ursolic acid: biological functions and application in animal husbandry

**DOI:** 10.3389/fvets.2023.1251248

**Published:** 2023-10-25

**Authors:** Guanhui Liu, Peng Qin, Xinying Cheng, Lifei Wu, Ruoning Wang, Wei Gao

**Affiliations:** ^1^School of Life Sciences and Food Engineering, Hebei University of Engineering, Handan, China; ^2^Chenguang Biotechnology Group Handan Co., Ltd., Handan, China; ^3^Hebei Plant Extraction Innovation Center Co., Ltd., Handan, China; ^4^Hebei Province Plant Source Animal Health Products Technology Innovation Center, Handan, China

**Keywords:** ursolic acid, anti-inflammatory, antioxidative, antimicrobial, hepatoprotective, anticancer, antiviral activity, animal husbandry

## Abstract

Ursolic acid (UA) is a plant-derived pentacyclic triterpenoid with 30 carbon atoms. UA has anti-inflammatory, antioxidative, antimicrobial, hepato-protective, anticancer, and other biological activities. Most studies on the biological functions of UA have been performed in mammalian cell (*in vitro*) and rodent (*in vivo*) models. UA is used in animal husbandry as an anti-inflammatory and antiviral agent, as well as for enhancing the integrity of the intestinal barrier. Although UA has been shown to have significant *in vitro* bacteriostatic effects, it is rarely used in animal nutrition. The use of UA as a substitute for oral antibiotics or as a novel feed additive in animal husbandry should be considered. This review summarizes the available data on the biological functions of UA and its applications in animal husbandry.

## 1. Introduction

**Figure 1:**
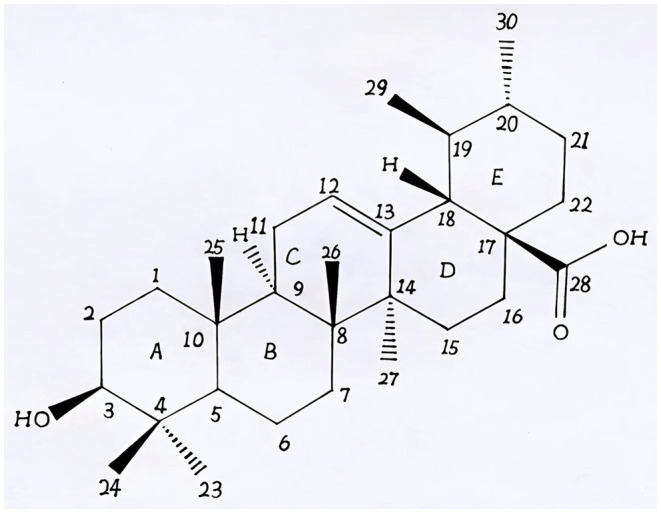
The chemical structure of ursolic acid.

Natural products derived from plants, including polyphenols, flavonoids, terpenoids, essential oils, and alkaloids, have many biological and pharmacological properties, including antibacterial, antiviral, anticancer, anti-inflammatory, anti-diabetic, and hepato-protective activities (1). Ursolic acid (UA) is a natural plant product with the chemical formula of C_30_H_48_O_3_ (Figure 1). It is found in the stem bark, leaves, and peel of Chinese herbs and fruits and has been shown to have a wide range of pharmaceutical properties (Table 1). UA contents vary in different plants, parts of plants, and the sources of plants (15), ranging from 0.091 to 1.58% in five different species of the *Lamiaceae* family (*Rosmarinus officinalis* L., *Salvia officinalis* L., *Satureja montana* L., *Salvia sclarea* L., and *Salvia glutinosa* L.) (16), reaching 49.7% in apple pomace and 22.7% in rosemary leaves (17). UA contents tend to be highest in flowers and leaves, and lower in stems and rhizomes (18). Details on UA contents and extraction methods from different plants are summarized in Table 2.

**Table 1:** The sources and pharmaceutical properties of ursolic acid.

Ursolic acid sources	Plant parts used	Experimental treatment	*in vivo* or *in vitro*	Bioactivities	Bibliography
*Diospyros dendo*	Dried leaves	*Pseudomonas aeruginosa* PA01	*in vitro*	Antibacterial activity	(2)
*Rosmarinus officinalis*	Dried leaves	Oral, 100 mg/kg/day of *R. officinalis* extract, for 15 days, BALB/c male mice	*in vivo*	Pro-neurogenic effects	(3)
*Ligustrum lucidum*	Fruit	Ovariectomized rats fed with diet containing the fruit of *Ligustrum lucidum Ait* (18.8 g/kg) for 6 weeks	*in vivo*	Osteoprotective effects	(4)
*Ligustrum lucidum*	Leaves	The methanol extract from *Ligustrum* plant leaves (0.1, 0.25, 1 g/kg) were orally administered to mice	*in vivo*	Anti-inflammatory and analgesic effects	(5)
*Melissa officinalis L.*	Aerial parts	The two breast cancer cell lines, MCF-7 and MDA-MB-231	*in vitro*	Anti-cancer	(6)
*Malus pumila*	Peels	Tumorigenic highly metastatic *ras/myc* serum-free mouse embryo cells, UA (2.5–10 μM)	*in vitro*	Anti-tumor	(7)
*Bursera cuneata*	Aerial parts	Mouse ear edema	*in vivo*	Anti-inflammatory and antihistaminic activity	(8)
*loquat*	Leaves	Oral, 50 mg/kg/day for 15 days, diabetic db/db mice	*in vivo*	Antioxidant and anti-inflammatory effects	(9)
*Ocimum gratissimum L. (Lamiaceae)*	Leaves	Red blood cells	*in vitro*	Anti-sickling activity	(10)
*Hawthorn (Crataegus* spp.*)*	Bark, leaves, berries	Oral,100 mg/kg/day for 28 days, rat	*in vivo*	Hypolipidemic and hepatoprotective effects	(11)
*Vitex negundo Linn*.	Fresh leaves	200 μg/mL of petroleum ether extract and chloroform extract, *Mycobacterium tuberculosis H37RV*	*in vitro*	Antitubercular activity	(12)
*Ixora coccinea Linn*	Flowers	Lung cancer (A549 and H460) and leukemia cell lines (K562, Lucena, HL60, and Jurkat)	*in vitro*	Inhibits the proliferation of cancer cells	(13)
*Eucalyptus tereticornis Sm.*	Leaves	The prediabetic mice were performed by intraperitoneal injection of the extract of *E. tereticornis* leaves (300 mg/kg b. w.)	*in vivo*	Anti-obesity and anti-diabetes effects	(14)

**Table 2:** Ursolic acid contents and extraction methods from different plants.

Ursolic acid sources	Regions	Extraction method	Amount of ursolic acid	Bibliography
*Aralia chinensis*	China	Accelerated solvent extraction (95% ethanol)	0.29%	(19)
*Eriobotrya japonica Thunb*	China	Accelerated solvent extraction (95% ethanol)	0.69 mg/g	(19)
*Lavandula stoechas* L.	Türkiye	Maceration (methanol-dichloromethane 1: 1)	22.2 mg/g	(20)
*Lavandula stoechas* L.	Türkiye	Maceration (methanol)	12 mg/g	(20)
*Lavandula stoechas* L.	Türkiye	Maceration (Ethanol)	13.2 mg/g	(20)
*Lavandula stoechas* L.	Türkiye	Maceration (Acetone)	17.5 mg/g	(20)
*Lavandula stoechas* L.	Türkiye	Maceration (Ethyl acetate)	19.7 mg/g	(20)
*Lavandula stoechas* L.	Türkiye	Maceration (Diethyl ether)	10.6 mg/g	(20)
*Lavandula stoechas* L.	Türkiye	Maceration (Chloroform)	18.5 mg/g	(20)
Leaves of *Melissa officinalis* L.	The western area of Romania	Accelerated solvent extraction (70% ethanol)	3.58 mg/g	(6)
Leaves of *Melissa officinalis* L.	The western area of Romania	Accelerated solvent extraction (96% ethanol)	6.10 mg/g	(6)
Leaves of *Melissa officinalis* L.	The western area of Romania	Accelerated solvent extraction (80% methanol)	11.23 mg/g	(6)
Leaves of *Ilex aquifolium* L.	North-East Corsica, France	Soxhlet apparatus (hexane and dichloromethane)	1.3%	(21)
Fruits of *Chaenomeles speciosa*	China	Accelerated solvent extraction (80% ethanol)	0.164–0.340 mg/g	(22)
Leaves of *Catharanthus roseus*	India	Soxhlet apparatus (hexane, chloroform, and methanol)	–	(23)
Leaves of *Neolamarckia cadamba*		Sono-Maceration (ethyl acetate)	27.5 mg/g	(24)
Leaves of *Rosmarinus officinalis*	Lithuania	Ultrasound assisted extraction (90% ethanol)	15.8 mg/g	(25)

In terms of biopharmaceutical classification, UA is a Class IV compound (“low” solubility and “low” permeability) (26). The clinical application of UA is limited because of its poor bioavailability, together with low intestinal permeability and solubility. UA can be used as a component in the vesicle-like nanocarrier system because of its small size. Moreover, UA can act not only as an anticancer additive in the nanocarrier system, but it also shows synergy with other drugs, indicating further advantages in medical treatments (27). Previous research has suggested several strategies, including complexation with hydrophilic cyclodextrins (28), structure modification (29), nanotechnology (30), and creating a supramolecular coamorphous system of UA with piperine (31), to overcome these limitations.

UA has been proposed as a candidate drug for treating various cancers (32–35), inflammatory diseases (36), diabetes (37), Parkinson’s disease (38), Alzheimer’s disease (39), and liver-related diseases in humans. However, studies have only been performed in mouse models, and there are few studies on the potential applications of UA in farm animals. This review aims to discuss the biological activities of UA and investigate the feasibility of using UA in animal husbandry.

## 2. Beneficial effects of UA

### 2.1. Anti-inflammatory effects

The close association between inflammation and many diseases, such as Parkinson’s disease, osteoarthritis, cardiovascular events, diabetic nephropathy, cancer, and influenza infection, is well known (40). Previous *in vivo* and *in vitro* studies have shown that UA acts against both endogenous and exogenous inflammatory stimuli, with favorable anti-inflammatory effects. UA (25 mg/kg body weight, oral) can prevent the degeneration of dopaminergic neurons in 1-methyl-4-phenyl-1,2,3,6-tetrahydropyridine-induced Parkinsonian mice (41). UA has been shown to significantly suppress xylene-induced ear edema *in vivo* as well as protect against lipopolysaccharide (LPS)-induced acute kidney injury by blocking the Toll-like receptor/myeloid differentiation primary response 88 pathway *in vitro* (42). In addition, UA can alleviate osteoarthritis by inhibiting the nuclear factor kappa B/NOD-like receptor protein-3 (NF-κB/NLRP3) inflammasome pathway (43). Furthermore, UA, at concentrations of 160 and 320 μg/mL, could inhibit inflammatory responses via the phosphatidylinositol 3-kinase/protein kinase B (PI3K/Akt) and NF-κB signaling pathways, reducing the viability of breast cancer cells (44). Treatment with 50 mg/kg UA in combination with caprylic acid (60 mg/kg) was found to decrease the levels of inflammatory cytokines such as tumor necrosis factor-alpha (TNF-α), interleukin-1-beta (IL-1β), to reverse pentylenetetrazole-induced seizure-like symptoms in a zebrafish model (45).

When used at a concentration of 20 μg/mL, UA, in an influenza A virus (IAV)-treated A549 cell model, resolved both cell injury and the inflammatory response (46). Zhou and Wink (47) also revealed that UA can play an anti-inflammatory role by inhibiting NF-κB nuclear translocation, thereby reducing the expression of inflammation-related factors, such as TNF-α, cyclooxygenase-2, and inducible nitric oxide synthase (iNOS). UA derivatives have also been found to be effective in treating inflammation. In a two-stage skin carcinogenesis mouse model, two weeks of treatment with UA (2 μmol) or its synthetic derivatives significantly inhibited the expression of pro-inflammatory genes, such as interleukin-1-alpha (IL-1α), IL-1β, IL-6, and IL-23. Moreover, a few UA derivatives, such as corosolic acid and 3-epi-corosolic acid, were found to have stronger anti-inflammatory activities than UA (48). Similarly, another UA derivative, β-D-glucopyranosyl ester, was also found to have anti-inflammatory activity (49). Overall, previous studies have established that UA and its derivatives regulate the development of inflammation, and hence may be useful for treating inflammation-related diseases.

### 2.2. Antioxidant activity

Oxidative stress results from excessive production of reactive oxygen species (ROS) in cells and tissues, which further leads to various inflammation-associated diseases (50). The *in vitro* antioxidant activity of UA was evaluated by inhibiting 2,2-diphenyl-1-picryl-hydroxyl at an inhibitory concentration (IC_50_) of 59.7 ± 1.0 μg/mL (51). Administration of UA (25 and 50 mg/kg of body weight/day, intragastrically) for 6 weeks was found to reduce CCl_4_-induced nephrotoxicity, demonstrating the antioxidant activity of UA and its ability to inhibit the phosphorylation of transcription 3 (STAT3)-NF-κB pathway (52). In human lymphocytes and the hamster V79 lung fibroblast cell line, UA was demonstrated to be a natural antioxidant that prevents DNA damage caused by hydrogen peroxide, causing a 50% decrease in cell viability at 224.85 mM (53). Furthermore, UA has been suggested as a potential candidate for the treatment and prevention of oxidative stress-mediated diseases (54), including neurodegenerative diseases in mouse models (55), obesity/diabetes and cardiovascular diseases in mice, skin carcinogenesis in mouse epidermal cells (56), liver disease in mice with carbon tetrachloride (CCl_4_)-induced liver fibrosis (57), and osteoporosis in MG-63 cells (58).

UA plays a vital role in the injury caused by cerebral ischemia in mice by activating the nuclear factor-erythroid 2-related factor 2 (Nrf2) pathway (59). Nrf2, encoded by the gene *NFE2L2*, is a regulatory factor of phase II antioxidant enzymes, protecting the body from oxidative stress and inflammation. Nrf2^(−/−)^ mice showed obvious symptoms of neurodegeneration and oxidative stress, which were significantly reduced by intraperitoneal injection of UA (100 mg/kg), demonstrating the neuroprotective effects of UA (55). UA (50 mg/kg/day, 6 weeks, oral gavage) was also shown to attenuate CCl_4_-induced hepatic oxidative damage and inflammation by increasing the expression levels of NAD(P)H:quinone-oxidoreductase-1, glutathione S-transferases, heme oxygenase-1, B-cell lymphoma-2 (Bcl-2), and nuclear Nrf2 (57). In a *Caenorhabditis elegans* model used for the evaluation of neurological drugs, UA (100 μM) showed antioxidant activity by upregulating the expression of peroxiredoxin-2 and skn-1 transcription factor (which corresponds to human Nrf2); this also led to an anti-depressant effect (60). Dietary administration of 0.1% UA (for 8 weeks) attenuated the tumor growth of transplanted VCaP (human prostatic cancer cell) xenografts in immunodeficient mice, together with epigenetic CpG methylation reprogramming, suggesting potential applications for the treatment/prevention of human prostate cancer (61). Treatment of JB6 P+ mouse epidermal cells with 2.5 μM UA increased Nrf2 expression by altering the methylation status of the Nrf2 promoter, thus inhibiting the development of skin cancer (56). In cisplatin-resistant HepG2/DDP cells, UA, as a natural adjuvant, increased the sensitivity of hepatocellular carcinoma cells to cisplatin through the Nrf2/antioxidant reaction element (ARE) signaling pathway, thus exhibiting anticancer effects (62). In conclusion, the results of various studies indicate that Nrf2 may be the target of the biological activity of UA.

### 2.3. Anticancer activity

**Figure 2:**
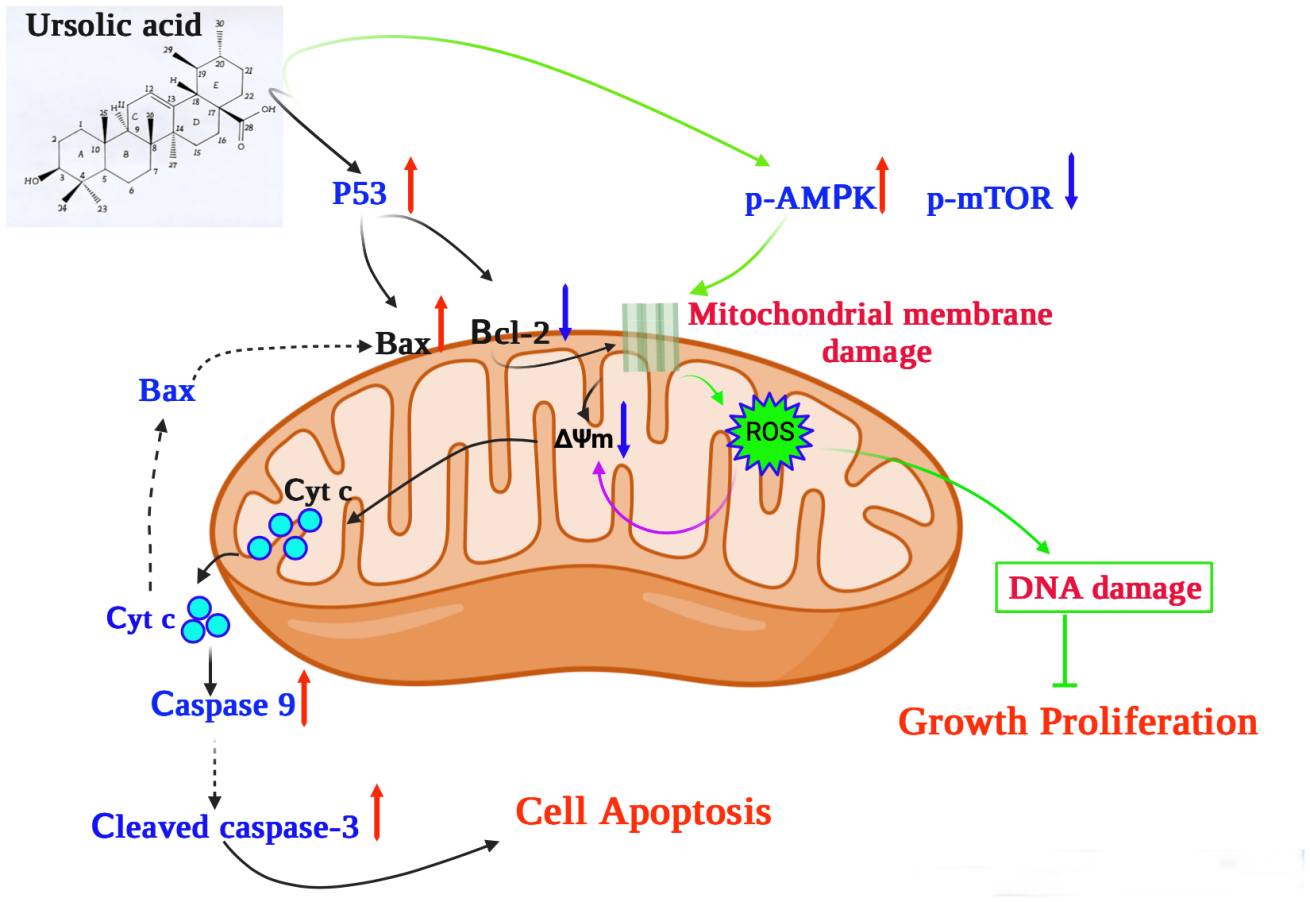
The potential mechanism underlying the anticancer effects of ursolic acid (64–68). UA upregulates and activates the expression of the cell tumor antigen p53 and the AMP-activated protein kinase (AMPK) signaling pathway, respectively, together with suppression of the mammalian target of rapamycin (mTOR) pathways and downregulation of the apoptosis regulator Bcl-2. UA damages the mitochondrial membrane, decreasing the mitochondrial membrane potential ΔΨm, which induces cytochrome c release from the mitochondria to the cytosol, activating the caspase proteins and leading to apoptosis. In addition, UA treatment significantly increases the production of mitochondrial ROS, reducing both DNA synthesis and cell proliferation. UA, ursolic acid; p-AMPK, phosphorylated AMP-activated protein kinase; mTOR, mammalian target of rapamycin; Bcl-2, B-cell lymphoma 2; ROS, reactive oxygen species; Cyt C, Cytochrome C; ∆Ψm, mitochondrial membrane potential.

In terms of its anticancer activity, UA is mainly associated with apoptosis and death of cancer cells. Mitochondria are essential for cell respiration and oxidative phosphorylation, and mitochondrial damage leads to apoptosis (63). UA exerts its anticancer effect by activating mitochondrial-dependent signaling pathways (Figure 2).

For example, UA upregulates and activates the expression of the cell tumor antigen p53 and the AMP-activated protein kinase (AMPK) signaling pathway, respectively, and downregulates the expression of the apoptosis regulator Bcl-2 (64). In a previous study, UA was shown to induce apoptosis of human breast adenocarcinoma (MCF7), osteosarcoma (U2OS), cervical adenocarcinoma (HeLa), and colorectal cancer (HCT116) cells after 24 h of treatment, with lethal dose 50 (LD_50_) values of 12.8 μM, 7.7 μM, 16.8 μM, and 19 μM, respectively (69). Furthermore, UA (20 μM) could inhibit the expression of AKT mRNA, thereby activating the AMPK signaling pathway to promote autophagy and apoptosis (65). Furthermore, in the presence of 30 μM UA for 48 h, the proliferative activity of human lung cancer cells (A549 and H460) decreased significantly, whereas the expression of caspases 3 and 9 increased, further increasing the levels of the apoptosis-related protein Bax and decreasing those of the anti-apoptotic protein Bcl-2 (66). These results suggest that UA can induce apoptosis through caspase-related signaling pathways. Through the action of UA, AMPK is activated and the mammalian target of the rapamycin (mTOR) signaling pathway is suppressed, thus controlling protein synthesis and cell growth (66). In addition, structurally modified UA may also activate non-apoptotic cell death pathways associated with autophagosome and lysosome accumulation (67). UA also showed therapeutic effects on drug-resistant cancer cell lines. *In vitro* experiments confirmed that 48 h of UA treatment (16 μM) could reduce the adhesion and infiltration of the human breast cancer adriamycin-resistant cell line MCF-7/ADR to human umbilical vein endothelial cells (HUVECs) and could also reduce migration in MCF-7/ADR cells (68). UA induced cellular DNA damage and initiated G0/G1 phase arrest in embryonic cancer cells, and could be a candidate for inhibiting the recurrence of cancer (70).

### 2.4. Hepato-protective activity

When C57BL/6 mice were fed a high-fat diet for 15 weeks, accompanied by oral administration of UA, 80 mg/kg of UA could significantly reduce the total cholesterol (TC) and triglyceride (TG) levels in the liver and plasma, effectively relieve liver steatosis and reduce the number of epididymal fat cells. Meanwhile, *in vitro* experiments also confirmed that UA (20 μM) significantly reduced TC (37.2%) and TG (50.4%) contents in HepG2 cells and upregulated P-AMPK protein expression (71). Therefore, UA may reduce the lipid content of cells and inhibit lipid synthesis by activating the AMPK signaling pathway. UA improves the richness of beneficial intestinal flora through inhibition of the NOX4/NLRP3 inflammasome pathway, thus alleviating liver fibrosis caused by CCl_4_ (72). UA can also reduce the activation of mitogen-activated protein kinases (JNK, p38 MAPK, ERK) and inactivate the immunoregulatory transcription factor NF-κB in the liver after treatment with CCl_4_, thereby relieving CCl_4_-induced inflammation (73).

**Figure 3:**
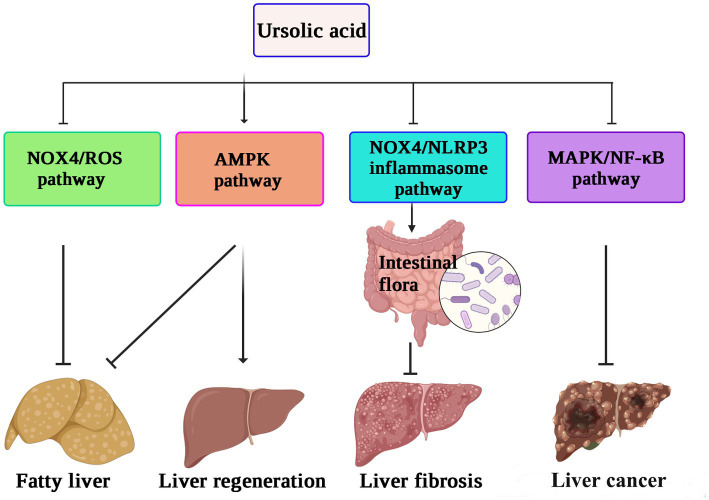
The hepatoprotective mechanisms of ursolic acid (71–75). UA suppresses the NOX4/ROS pathway to relieve nonalcoholic fatty liver disease, activates the AMPK pathway to enhance liver regeneration, and inhibits the MAPK/NF-κB pathway to decrease the proliferation of cancer cells. In addition, the protective effect of UA against liver fibrosis may be related to reducing inflammation and oxidative stress levels or regulating intestinal flora by downregulation of the NOX4/NLRP3 pathway. UA, ursolic acid; AMPK, AMP-activated protein kinase; NOX4, NADPH oxidase 4; MAPK, mitogen-activated protein kinase. NLRP3, NOD-like receptor thermal protein domain associated protein 3; ROS, reactive oxygen species; NF-κB, nuclear factor kappa-B.

*In vitro* experiments have also shown that UA can reverse the progression of liver fibrosis by inhibiting the activation of the NADPH oxidase (NOX)/ROS signaling pathway in hepatic stellate cells (74). UA protects the intestinal barrier by inhibiting the inflammatory factor TNF-α and increasing the expression of tight junction proteins and antimicrobial peptides. A 16S rRNA gene sequencing study also confirmed that UA treatment increased the abundance of beneficial bacteria, such as *Firmicutes*, *Lactobacillus,* and *Bifidobacterium*, thus alleviating the liver fibrosis process (75). Speculatively, the protective effect of UA on the liver may be related to reducing inflammation and oxidative stress levels or regulating intestinal flora. The possible mechanism underlying the role of UA in hepatoprotection against liver fibrosis, inhibiting the proliferation of cancer cells, and reversing nonalcoholic fatty liver disease, thus promoting liver regeneration by different pathways, is shown in Figure 3.

### 2.5. Antibacterial activity

Antibiotic resistance and the emergence of superbugs have become serious public health problems in both human and veterinary medicine. To treat infections caused by multi-drug resistant pathogens, many different strategies have been used, including phages (76), antimicrobial peptides (77), metal nanoparticles (78), and plant-derived compounds--polyphenols (79), flavonoids (80), terpenoids (81), plant essential oils (82), and alkaloids (83). Traditional Chinese Medicine and Ayurveda have also been used in health care and disease prevention in ancient China and Egypt (84). UA, as a representative triterpenoid, showed significant antibacterial activity against the production of biofilm by *Escherichia coli* CFT073 (ATCC 700928), *Enterobacter cloacae* ATCC-BAA 2468, and *Pseudomonas aeruginosa* ATCC 25000 (81).

Although the antibacterial effects of plant extracts may not be as obvious as those of antibiotics, plant extracts can have synergistic effects when combined with antibiotics. UA showed a strong synergistic effect when combined with β-lactam antibiotics (ampicillin and benzacillin), increasing the sensitivity of *Staphylococcus aureus* and *Staphylococcus epidermidis* to antibiotics (85). UA (32 μg/mL) also displayed synergy with colistin against clinical isolates of *Klebsiella pneumoniae BC936* and *E. coli U3790* (86). Table 3 shows the MIC values of UA against different Gram-positive and Gram-negative pathogens. The MIC value of UA or its oleanolic acid isomer was 31.25 μg/mL against both *S. aureus* ATCC 6538 and methicillin-resistant *S. aureus*, whereas the MIC against *Listeria monocytogenes* ATCC 19115 was 250 μg/mL (91). UA is also known to show antifungal activity against *Cryptococcus neoformans* H99 with a MIC value of 250 μg/mL (92).

**Table 3:** MIC values of UA against different bacterial pathogens.

	Pathogens	Source	MIC value (μg/mL)	Bibliography
Gram-positive pathogens	MRSA ATCC 43300	Leaves of *Alstonia scholaris*	64	(87)
	MSSA ATCC 29213		16	
	*Enterococcus faecalis* ATCC 29212		1	
	*Bacillus cereus* ATCC 9139		8	
	*Listeria monocytogenes* ATCC 7644		2	
	MRSA (ATCC33591)	Leaves and twigs of *Vitellaria paradoxa*	16	(88)
	*Streptococcus sanguinis* ATCC 10556		128	(89)
	*Streptococcus gordonii* ATCC 10558		64	
	*Streptococcus mutans* UA159		256	
	*Streptococcus sobrinus* ATCC 6715		64	
	*Actinomyces viscosus* ATCC 15987		32	
	*Actinomyces naeslundii* ATCC 12104		16	
	*K. pneumoniae* ATCC 43816		400	(90)
	Four clinical isolates of MRSA		8	(88)
Gram-negative pathogens	*Escherichia coli* (ATCC 35150)	Leaves of *Alstonia scholaris*	>128	(87)
	*Salmonella enterica* (ATCC 13311)		>128	
	*Pseudomonas aeruginosa* (ATCC 27853)		>128	
	Four clinical isolates carbapenem-resistant *Klebsiella pneumoniae* strains		800	(90)

UA exposure led to a decrease in intracellular pH and ATP, downregulated the expression of four biofilm-related genes (*pgaA*, *luxS*, *wbbM*, and *wzm*), and inhibited biofilm formation of carbapenem-resistant *Klebsiella pneumoniae* (90). The antimicrobial activity of UA is stronger than that of tetracycline, with reductions of 50.5 and 12.7%, respectively, in staphylococcal membrane integrity (87). The MIC value of UA against *E. coli* (ATCC 25922), *K. pneumoniae* (ATCC10031), and *Shigella flexella* (ATCC12022) was 64 μg/mL. However, the MIC of UA against these bacteria was significantly reduced by modification of C-3 (hydroxyl) in UA to form UA ester analogs. Hence, the modified UA derivatives showed markedly increased antibacterial effects (51). Gram-negative bacteria contain an outer membrane, which is rich in LPS and phospholipids and prevents entry of UA into the bacterial cells, and hence the MICs of UA against Gram-negative bacteria are higher than that against Gram-positive bacteria (81).

UA kills bacteria by altering the structure of the bacterial cell, particularly by interfering with the cell membrane and adhesion proteins (93). UA also affects cell morphology and controls the expression of genes related to virulence factors, such as pili and fritillary (94). UA inhibits bacterial growth by reducing the ability of bacteria to adhere to host cells, as well as disrupting biofilm formation (95). In the presence of 10 mg/L UA from *Diospyros dendo* leaves/flowers, decreases of 72, 87, and 57% were observed in the biofilm biomass of *Escherichia coli* (ATCC 25404), *Pseudomonas aeruginosa*, and *Vibrio havii,* respectively, after culture for 24 h. Furthermore, 10 and 30 mg/L of UA induced the expression of genes related to chemotaxis (*cheA, motAB, tap,* and *tsr*). Overexpression of the *motAB* genes has been shown to cause increased bacterial activity, decreasing their stability in the biofilm environment and resulting in reduced biofilm formation (96). Experiments, such as measurement of bacterial glucosyltransferase activity, computer simulation, site-directed mutagenesis, and capillary electrophoresis, have revealed that UA competes for occupancy of the active site of glycosyltransferase, a key enzyme required for the synthesis of extracellular polymeric substances (EPS), secreted by *S. mutans,* thus inhibiting EPS formation and reducing the viability of *S. mutans* and the structural integrity of its biofilm (97). Therefore, *in vitro* results support the use of UA for the treatment of bacterial diseases. However, UA is rarely used to prevent or treat bacterial diseases in animal husbandry.

## 3. The side effects of UA

It has been reported that UA (5 mg/kg body weight, intraperitoneally) could inhibit the spermatogenesis in Wistar strain male albino rats (3 months old) (98). 50 μM UA purified from loquat (*Eriobotrya japonica*) also exerts the cytotoxicities against A549 (lung cancer cell line) and NTUB1 cells (human bladder cancer cell line) (99). In the acute and toxicity test, UA (21.5 g/kg body weight, oral) extracted from *Ledum pulastre* L. can destroy the nervous and digestive systems of mice, and the LD50 of the UA was 9.26 g/ kg (100).

## 4. Application of UA in livestock, poultry, and aquatic animals

At present, the application of UA in animal husbandry is limited. UA is mainly used to improve antioxidant capacity in broilers (101) and as a cryopreservative for porcine semen (102). UA is also used to treat bacterial pneumonia in calves (103) and improve intestinal immunity or treat viral diseases in fish, indicating potential applications in aquaculture (104).

Although the protective effect of UA against asthenozoospermia in rats by increasing sperm density and motility has already been established (105), its use as a cryopreservative for porcine semen has also been documented. As UA is a natural antioxidant, its addition (at a concentration of 1.6 mg/mL) to the frozen diluent of porcine sperm resulted in a significant improvement in the integrity of the mitochondrial membranes, plasma membranes, and acrosomes of the sperm (102). Supplementation of UA (400 mg/kg, purified from pine bark, and *Rosmarinus officinalis* L., *Aurantii fructus immaturus,* and *Eucommla ulmoides* leaves) for 42 days resulted in a significant reduction in elimination and mortality rates of the sperm, while the malondialdehyde content was also decreased due to an increase in the activity of superoxide dismutase (101). UA or UA derivatives have also been used as anti-microbial and anti-influenza drugs (106–109), and feed additives for the improvement of intestinal health and immunity in livestock and poultry (110–112).

In addition, using *in vitro* studies, UA has been demonstrated as a promising candidate for the treatment of bovine endometritis (113). Furthermore, it was found that when UA was used at a concentration of 20 μM, it provided protection in an LPS-induced model of endometrial cell inflammation. Notably, reductions in the expression of several pro-inflammatory cytokines, such as IL-6, TNF-α, and IL-1β, and, particularly, the NF-κB signaling pathway, were observed (113). Treatment with UA can result in the suppression of IL-17A expression in the lungs of newborn calves, and UA has also been demonstrated to have some therapeutic effect on bovine bacterial pneumonia caused by *Mannheimia haemolytica* (103). UA treatment (at a concentration of 1 ppm) was also found to be significantly useful in relieving renal malformations (atrophic glomeruli and curvature defects of the anterior renal ducts) caused by aristolochia acid in zebrafish embryos (114). A previous study demonstrated the useful therapeutic effects of sage and lemon verbena, which are also rich in triterpenoids. These molecules can increase the number of intestinal goblet cells, change the glycosylation properties of mucin agglutinin, and enhance intestinal immunity in juvenile gilthead seabream (*Sparus aurata*) (115).

Although the water insolubility of UA limits its clinical application, structurally modified UA shows antiviral activity. UA derivatives (esterification of the C-17 carboxyl or conversion of the carboxyl into amide) also exhibited a strong anti-H5N1 activity (116). Furthermore, 3-o-β-UA as well as UA’s ester equivalents showed antiviral activity against porcine reproductive and respiratory syndrome viruses *in vitro* (117). UA is one of the main components of *Prunella vulgaris L.*, and hence showed antiviral activity against hematopoietic necrosis virus (IHNV) in rainbow trout (*Oncorhynchus mykiss*). When UA was injected intraperitoneally, a reduction in the expression levels of IHNV glycoprotein mRNA (in the spleen) was observed on day 1 after viral infection (118). In addition, 10% UA obtained from *Lippia citriodora* and *Salvia officinalis* could be used as an effective additive to improve the growth performance and feed conversion ratio and provide immune protection after LPS treatment in juvenile gilthead seabream (*Sparus aurata*) (119). UA could also effectively inhibit *Micropterus salmoides rhabdovirus* (MSRV) replication (IC50 = 5.55 μM) *in vitro* and increase the mortality rate by 12.5% in MSRV-infected largemouth bass (104). This indicates that UA can be considered as an alternative anti-MSRV agent in aquaculture. An extract of *O. sanctum* containing UA was found to have potential anti-influenza activity (120), and the juice from fresh *O. sanctum* leaves exhibited antibacterial activity due to the inhibition of the expression of extended-spectrum β-lactamase (ESBL) enzymes produced by *E. coli* (121). Based on these results, it can be predicted that UA has the potential to be used as an antiviral drug or antibiotic substitute in animal husbandry.

## 5. Insights and future directions

**Figure 4:**
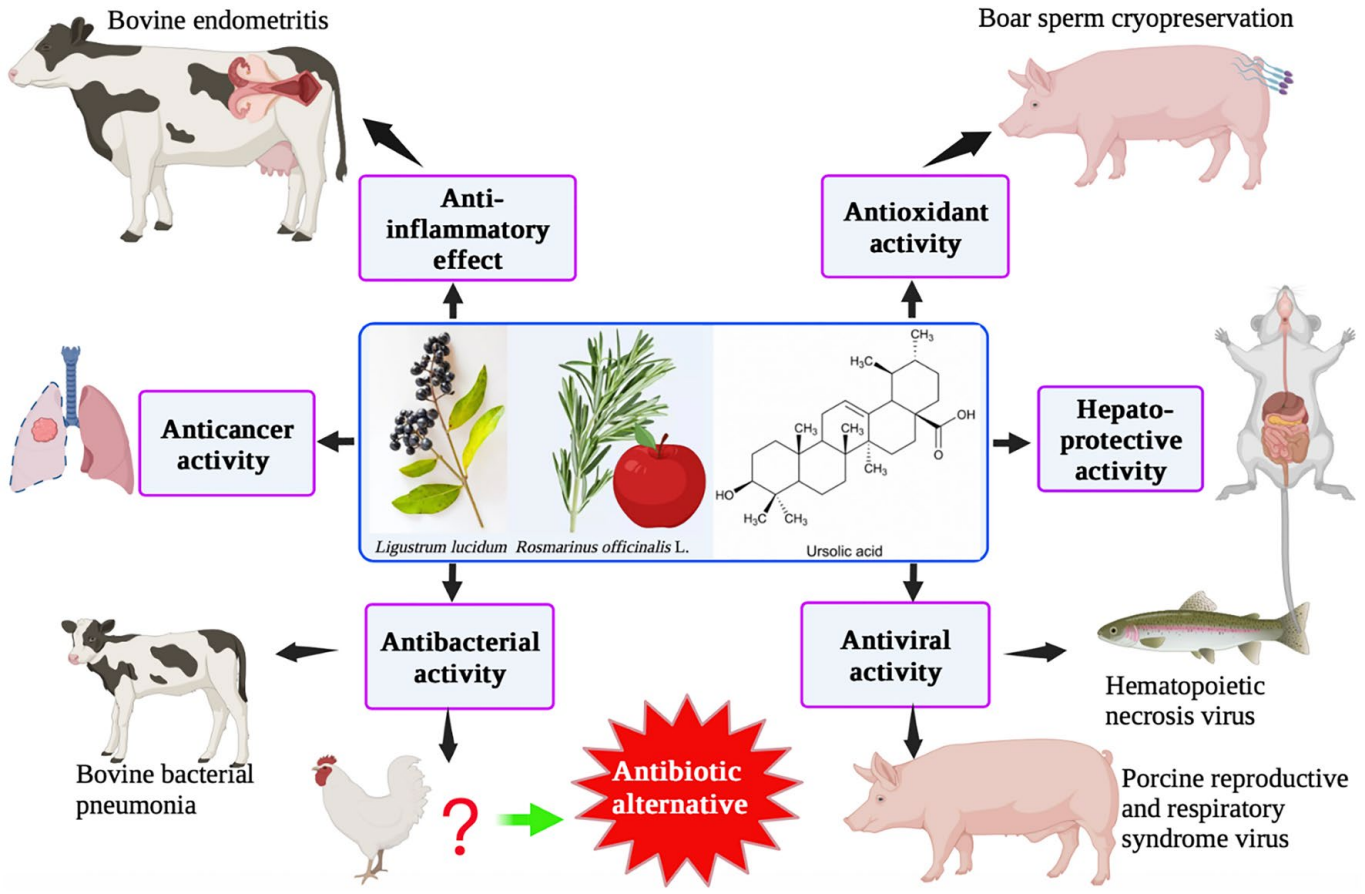
Ursolic acid: biological functions and applications in animal husbandry.

UA has a typical triterpenoid structure and thus has specific pharmacological characteristics. UA is found in a variety of fruits, spices, and medicinal herbs, such as *Lavandula stoechas*, apple peel, rosemary, and ligustrine. Numerous investigations have demonstrated that UA has a variety of biological activities, including anti-inflammatory, antioxidant, anti-cancer, and hepatoprotective effects, using both *in vitro* and *in vivo* animal model experiments (Figure 4). UA is mainly used in animal husbandry to improve intestinal immunity and for its anti-inflammatory and antiviral activities. Based on its bacteriostatic effects, UA has the potential to be used as a therapeutic agent in farmed species to reduce the use of chemotherapeutics and enhance the move toward sustainability.

However, although ultra-pure and high-dose UA has specific beneficial effects, the economic cost of UA production from plants requires consideration for its widespread use in livestock and poultry production. Furthermore, the toxicology and pharmacokinetics (bioavailability) of UA require further investigation.

## Author contributions

GL, PQ, and XC wrote sections of the manuscript. LW and RW made the figures. WG provided the topic of this article. All authors contributed to the article and approved the submitted version.

## Figures and Tables

**Table:** 

UA	ursolic acid
LPS	lipopolysaccharide
NF-κB	nuclear factor kappa B
NLRP3	NOD-like receptor protein-3
PI3K	*phosphatidylinositol 3-kinase*
IAV	influenza A virus
TNF-α	tumor necrosis factor-alpha
IL-1α	interleukin-1-alpha
IC_50_	inhibitory concentration 50
LD_50_	lethal dose 50
STAT3	phosphorylation of transcription 3
Nrf2	nuclear factor-erythroid 2-related factor 2
CCl_4_	carbon tetrachloride
Bcl-2	B-cell lymphoma-2
ARE	antioxidant reaction element
AMPK	AMP-activated protein kinase
HUVECs	human umbilical vein endothelial cells
TC	total cholesteroland
TGs	triglycerides
IHNV	hematopoietic necrosis virus
ESBL	extended-spectrum beta-lactamase
